# Designing Functionalized Polyelectrolyte Microcapsules for Cancer Treatment

**DOI:** 10.3390/nano11113055

**Published:** 2021-11-13

**Authors:** Daria Kalenichenko, Galina Nifontova, Alexander Karaulov, Alyona Sukhanova, Igor Nabiev

**Affiliations:** 1 Laboratoire de Recherche en Nanosciences, LRN-EA4682, Université de Reims Champagne-Ardenne, 51100 Reims, France; daria.kalenichenko@univ-reims.fr (D.K.); galina.nifontova@univ-reims.fr (G.N.); 2 Laboratory of Nano-Bioengineering, Institute for Physics and Engineering in Biomedicine (PhysBio), National Research Nuclear University MEPhI (Moscow Engineering Physics Institute), 115409 Moscow, Russia; 3 Department of Clinical Immunology and Allergology, Institute of Molecular Medicine, Sechenov First Moscow State Medical University (Sechenov University), 119146 Moscow, Russia; drkaraulov@mail.ru

**Keywords:** polyelectrolyte microcapsules, doxorubicin encapsulation, quantum dots, optical encoding, fluorescence imaging

## Abstract

The engineering of delivery systems for drugs and contrasting labels ensuring the simultaneous imaging and treatment of malignant tumors is an important hurdle in developing new tools for cancer therapy and diagnosis. Polyelectrolyte microcapsules (MCs), formed by nanosized interpolymer complexes, represent a promising platform for the designing of multipurpose agents, functionalized with various components, including high- and low-molecular-weight substances, metal nanoparticles, and organic fluorescent dyes. Here, we have developed size-homogenous MCs with different structures (core/shell and shell types) and microbeads containing doxorubicin (DOX) as a model anticancer drug, and fluorescent semiconductor nanocrystals (quantum dots, QDs) as fluorescent nanolabels. In this study, we suggest approaches to the encapsulation of DOX at different stages of the MC synthesis and describe the optimal conditions for the optical encoding of MCs with water-soluble QDs. The results of primary characterization of the designed microcarriers, including particle analysis, the efficacy of DOX and QDs encapsulation, and the drug release kinetics are reported. The polyelectrolyte MCs developed here ensure a modified (prolonged) release of DOX, under conditions close to normal and tumor tissues; they possess a bright fluorescence that paves the way to their exploitation for the delivery of antitumor drugs and fluorescence imaging.

## 1. Introduction

Finding efficient systems for the targeted delivery of drugs and contrast agents to tumor growth areas and the stimulus-sensitive release of both drugs and agents is essential; therefore, it is at the forefront of the development of theranostic agents for the combined diagnosis and treatment of tumors [1,2].

Polyelectrolyte microcapsules (MCs) are versatile polymer microcontainers formed from the interpolymer complexes of oppositely charged polyelectrolytes [3]. The main advantage of polyelectrolyte MCs as drug-delivery systems is their capacity for the controlled, stimulus-sensitive release of their functional components in response to a specific trigger, including physical (ultrasound, magnetic field, laser pulse, or optical radiation), chemical (the pH and ionic strength of the microenvironment or solvent polarity), and biochemical (receptors or target cells) triggers [4,5,6,7,8,9,10].

The technique of MC synthesis allows for controlling the size, structure and surface characteristics of the resultant capsules [11]. Specifically, nano- and microsized calcium carbonate (CaCO_3_) particles of the vaterite type (microbeads (MBs)) are widely used as a substrate for the formation of the polyelectrolyte shell [9,10,12,13,14,15]. These particles are biocompatible and biodegradable [16,17,18,19]; their porous matrix structure is characterized by a large specific surface that ensures the adsorption of both polymers (in the course of microcapsule fabrication) and drugs or other biologically active substances [14,15,17,18], which makes them also promising as core-type carriers [17,18,19]. The application of the polyelectrolyte shell onto the surface of these cores yields core/shell MCs, and the subsequent dissolution of the cores results in the formation of soft shell-type MCs [14,20]. The microcapsules obtained using calcium carbonate microparticles are typically from 0.5 to 6.0 μm in size and are spherically shaped [7,12,14,17,20].

The method of layer-by-layer adsorption of polyelectrolytes used for the formation of the polymer shell allows magnetic [21,22] and fluorescent semiconductor [10,13,23] nanoparticles and other fluorescent labels [24] to be incorporated into the shell, thus ensuring magnetic resonance or fluorescence imaging of the MCs. These polyelectrolyte structures can be efficiently optically encoded with fluorescent semiconductor nanocrystals (quantum dots (QDs)) [23,25]. QDs have unique optical characteristics, including a wide absorption spectrum, a narrow, symmetrical fluorescence spectrum, and bright fluorescence, the lifetime of which exceeds that of the traditionally used organic fluorescent labels [26,27]. The incorporation of QDs into the polymer shell also weakens their potential toxic effect on live cells [4,28].

The MCs can be functionalized with high-molecular-weight compounds, including capture molecules for the specific recognition of target tumor cells (e.g., monoclonal anti-HER2, anti-EGFR antibodies, bispecific anti-EGFR antibodies [4,10,29]), short interfering RNA recognizing programmed cell death protein 1 mRNA [30], and low-molecular-weight antitumor agents (e.g., gemcitabine, 5-fluorouracil, phthalocyanines, and doxorubicin [6,15,31,32]). Doxorubicin (DOX) is the most commonly used first-line drug for a number of malignant tumors, such as ovarian, breast, and prostate cancers, as well as lymphomas and leukemias [33]. In addition, DOX is fluorescent due to the central anthracycline chromophore group, which makes it an attractive functional component to be used as a fluorescent label for the imaging of theranostic agents and *in vitro* monitoring of DOX release [34]. DOX encapsulation ensures its modified release, decreases its cytotoxic effect and allows the imaging of MC interaction with tumor cells [12,17].

The effective incorporation of doxorubicin into delivery systems, including polyelectrolyte MCs, is a challenging task due to the hydrophilicity of the DOX hydrochloride salt and amphiphilicity of the DOX base, respectively. Various approaches can be used to incorporate DOX into the MCs, such as spontaneous loading [3] and encapsulation at the stage of synthesis of the carrier using, e.g., the emulsion method [35], both of which approaches are widely used for loading DOX into carriers. However, alternative methods of DOX incorporation not requiring the preparation of an emulsion or the use of organic solvents and special equipment for dispersing are more promising for the development of approaches to the encapsulation of low-molecular-weight substances.

The versatility of layer-by-layer technology allows for multiple functionalization approaches that can be used at different stages of MC preparation. In the present study, we have designed both polyelectrolyte-coated MBs and polyelectrolyte MCs, to demonstrate the possibility of their use as delivery systems providing modified drug release and fluorescent imaging. This study was aimed at designing polyelectrolyte MCs and developing approaches to their functionalization with QDs that are water-solubilized using polyethylene glycol (PEG) derivatives, as well as using DOX as a model antitumor agent that contains hydrophilic moieties. Here, we describe an approach to obtaining size-homogeneous MBs and MCs with a mean size of 2–3 μm and different structures (core/shell and shell types). We present the data on the modification of the synthetized small-sized MCs with QDs and DOX, as well as the results of the primary characterization of these MBs and MCs obtained according to optimized protocols (their shape, size, surface charge, fluorescence characteristics, and release of the active principle at the pH values characteristic of normal and tumor tissues).

## 2. Materials and Methods

### 2.1. Synthesis of CaCO_3_ Microbeads

The CaCO_3_ MBs were obtained by crystallization [13,16]. Then, 7.5 mL of 0.33 M Na_2_CO_3_ (Merck Group, Sigma-Aldrich, Saint-Quentin-Fallavier, France) and 7.5 mL of 0.33 M CaCl_2_ (Merck Group, Sigma-Aldrich, Saint-Quentin-Fallavier, France) were mixed with equivalent volumes of a 44 wt % aqueous solution of glycerol (Merck Group, Sigma-Aldrich, Saint-Quentin-Fallavier, France), added to the reaction mixture as a thickening agent [12,15,16]. The Na_2_CO_3_ and CaCl_2_ solutions were preliminarily passed through filters with a pore size of 0.22 μm (Merck Group, Sigma-Aldrich, Saint-Quentin-Fallavier, France). The mixture was stirred for 60 min at 500 rpm using a magnetic bar stirrer. After that, the stirring was stopped, and the mixture was incubated at room temperature for 10 min. The precipitated MBs were washed free of excess thickening agent four times by successively centrifuging the suspension at 3000× *g* for 15 min and resuspending the pellet in a fresh portion of ultrapure water. After the final washing, the MBs were dried on Petri dishes in a drying oven at 90 °C overnight. 

### 2.2. Formation of Core/Shell and Shell Polyelectrolyte Microcapsules

The microcapsules of the core/shell (MB(+8L)) and shell (MC(8L)) types were obtained by means of layer-by-layer adsorption of oppositely charged polyelectrolytes, the polycation poly(allylamine hydrochloride) (PAH) and the polyanion poly(sodium 4-styrenesulfonate) (PSS) [12,15,23], onto the surface of CaCO_3_ MBs being used as a substrate. Initially, ~10^8^ MBs were dispersed in 0.5 mL of MilliQ water and sonicated on an ultrasound bath to obtain single particles in the suspension. Then, 0.5 mL of a 2 mg/mL PAH polycation (Mw ≈ 17,500 Da, Merck Group, Sigma-Aldrich, Saint-Quentin-Fallavier, France) solution in 0.5 M NaCl (Merck Group, Sigma-Aldrich, Saint-Quentin-Fallavier, France) was added to 0.5 mL of the suspension. The mixture was sonicated on an ultrasound bath for 60 s and incubated on an orbital shaker for 20 min at room temperature while stirring. Excess polyelectrolyte was removed by centrifugation at 1377× *g* for 3 min. To apply the next, polyanion layer, the MBs were resuspended in 0.5 mL of a 2 mg/mL PSS (Mw ≈ 70,000 Da, Merck Group, Sigma-Aldrich, Saint-Quentin-Fallavier, France) solution in 0.5 M NaCl, and the suspension was sonicated and incubated as described above. After the incubation, the particles were washed free of excess polyelectrolyte three times, each time with a fresh portion of ultrapure water, by centrifugation. The polyelectrolytes were applied onto the surface of the CaCO_3_ MBs in the following order: PAH/PSS/PAH/PSS/PAH/PSS/PAH/PSS. After the outermost layer was applied and the resultant core/shell MCs were washed, they were resuspended in a fresh portion of ultrapure water (0.5 mL) and used in subsequent experiments.

In order to obtain shell MCs(8L), the CaCO_3_ cores were dissolved by incubating the suspension in 0.5 M EDTA (pH 8.0). Approximately 10^7^ microparticles were placed into 5 mL of 0.5 M EDTA (pH 8.0) (Merck Group, Sigma-Aldrich, Saint-Quentin-Fallavier, France) and incubated for 4 h [10]. After that, the resultant MCs were sedimented by centrifugation, and the supernatant was replaced with ultrapure water. The washing of MCs to remove the products of core dissolution was repeated three times. After the last washing, the MCs were resuspended in 0.5 mL of ultrapure water.

### 2.3. Functionalization of the Microbeads and Microcapsules with Doxorubicin

The DOX-containing MBs were obtained by means of coprecipitation in the course of the microparticle synthesis [20]. Then, 1 mL of a 10 mg/mL DOX (Merck Group, Sigma-Aldrich, Saint-Quentin-Fallavier, France) solution was preliminarily added to a mixture containing 7 mL of 0.33 M CaCl_2_ and 7.5 mL of 44 wt % glycerol. The mixture was then placed onto a magnetic bar stirrer, and a mixture of 7 mL of 0.33 M Na_2_CO_3_ and 7.5 mL of 44 wt % glycerol was poured in under stirring at 500 rpm. The resultant mixture was stirred for 60 min in the dark, after which the stirring was stopped, and the incubation was continued for another 10 min at room temperature. The obtained DOX-containing MBs were washed free of residual reaction mixture two times. Then, the MBs were dried on Petri dishes in a drying oven at 90 °C overnight.

The synthesized MBs were used as a substrate for obtaining core/shell MCs (MBs(+8L)). The technique of layer-by-layer adsorption of polyelectrolytes described in Section 2.2 was used to apply the polyelectrolyte shell, according to the following scheme: MB/PSS/PAH/PSS/PAH/PSS/PAH/PSS/PAH/PSS.

DOX was loaded into the prepared MCs(8L) via diffusion, stimulated by a change in the permeability of the polyelectrolyte shell, which was caused by varying the pH of the dispersant and increasing the NaCl content of the MC(8L) microenvironment [8,35]. For this purpose, ~6 × 10^6^ MCs were placed into 0.5 mL of a 0.05 M phosphate buffer solution containing 0.5 M of NaCl and 16 μg of DOX. The MCs(8L) were incubated at 25 °C for 16 h under constant stirring. After the incubation, the MCs(8L) were centrifuged at 8609× *g* for 5 min, the supernatant was removed, and the pellet was finally resuspended in 0.5 mL of MilliQ water.

### 2.4. Modification of the Microcapsule Shell with Quantum Dots

CdSe/ZnS core/shell QDs with a fluorescence peak at a wavelength of 593 nm were used to optically encode the polyelectrolyte shell. The QDs were preliminarily transferred into the aqueous phase by means of ligand exchange and solubilized using thiol- and carboxyl-containing PEG derivatives, as described earlier [23,25]. They were embedded into the MC shell through adsorption onto the surface of the MBs with a preliminarily applied layer of the PAH polycation [13,36], to ultimately obtain the following sequence of layers: MB/PAH/PSS/PAH/QD/PAH/PSS/PAH/PSS.

Specifically, 3.8 × 10^5^ or 1.6 × 10^6^ QDs were added to 0.5 mL of a suspension of ~10^8^ DOX-free or DOX-containing MBs coated with a PAH/PSS/PAH shell, which was preliminarily sonicated on an ultrasound bath. The mixture was incubated at room temperature for 1.3 h under constant stirring and 30-s sonication every 20 min. When the QDs had been adsorbed, the microparticles were washed thrice with ultrapure water by centrifugation and resuspension. After that, the covering (PAH/PSS)_2_ layers were applied. To prepare hollow QD-encoded MCs, the calcium carbonate template was removed, as described in Section 2.2. Optical encoding of the MBs containing DOX was performed using the same procedure of layer-by-layer deposition of polyelectrolytes and QDs. The QD-encoded MCs with encapsulated DOX were prepared using primarily obtained QD-encoded MCs, which were subsequently incubated in a DOX solution under the conditions described in Section 2.3.

### 2.5. Characterization of Microbeads and Microcapsules with Different Structures

The shape and structure of the microparticles, as well as their size distribution, were analyzed by means of optical and fluorescence microscopies using an Axio Observer 3 microscope equipped with an XF115-2 FITC long-pass filter, which consisted of a 505DRLP dichroic filter, a 475AF40 excitation filter, and a 510ALP emission filter. For this purpose, 5 μL of an MB/MBs(+8L)/MC(8L) suspension was fixed in 10 μL of an 80% glycerol aqueous solution on a glass slide. The microscopic images were processed to estimate the size distribution using the *ZEISS Zen*, version 2.5 (blue edition); Software For Modular Image Acquisition, Processing and Analysis; Carl Zeiss: Jena, Germany, 2021, and *ImageJ*, version 1.53c; Software For Image Analysis; Rasband, W.S.: U. S. National Institutes of Health, Bethesda, Maryland, USA, 2020. 

Scanning electron microscopy (SEM) was performed using an SU8030 field emission gun scanning electron microscope (Hitachi, Tokyo, Japan). The powder of dried microbeads was applied onto a conducting carbon adhesive tape and scanned at an accelerating voltage of 3.0 kV, a working distance of 8.5–8.6 mm, and an emission current of 9000 nA.

The MB and MC surface charges, as well as the charges from the polyelectrolytes and functional components (DOX and water-soluble QDs), were estimated by Doppler microelectrophoresis using a Zetasizer NanoZS instrument (Malvern Panalytical, Palaiseau, France). 

### 2.6. Efficiency of the Incorporation of Quantum Dots into the Polyelectrolyte Shell

The efficiency of QD incorporation into the polyelectrolyte shell of the MCs was estimated as the difference between the initial amount of QDs used for encoding and the amount of QDs in the supernatant after adsorption. The amount of QDs adsorbed on the microparticle surface was calculated as:

(1) $${\mathrm{QD}}_{\mathrm{abs}}{=\mathrm{QD}}_{0}{\;-\;\mathrm{QD}}_{i},$$

where QD_0_ is the initial amount of QDs in the aliquot used for encoding and QD_i_ is the amount of QDs in the supernatant withdrawn after the encoding.

The number of QDs per MC was determined from the light absorption spectra using the following equation [23]:

(2) $$N_{\mathrm{QD}/\mathrm{MC}}=\frac{m_{\mathrm{QD}}{\;\times \;N}_{A}}{N_{\mathrm{MC}}{\;\times \;M}_{\mathrm{QD}}}=\frac{{A\;\times \;N}_{A}\;\times \;V}{N_{\mathrm{MC}}\;\times \;\varepsilon \;\times \;l},$$

where m_QD_ is the weight of QDs in the solution, N_A_ is the Avogadro number, N_MC_ is the number of MCs in the solution, M_QD_ is the QD molar weight, A is the optical density, V is the volume of the solution, l is the thickness of the absorbing layer, and ε is the QD molar extinction coefficient.

The amount of DOX loaded into MBs/MBs(+8L)/MCs(8L) was determined spectrophotometrically at the wavelength of the peak of DOX absorbance (485 nm) using a Spark™ 10M multimode microplate reader (Tecan, Männedorf, Switzerland). It was calculated as the difference between the initial amount of DOX used for encapsulation into the microcapsules and the residual amount of DOX left after the encapsulation. The encapsulation efficiency was calculated as:

(3) $$\mathrm{EE}=\frac{Q_{{\mathrm{DOX}}_{0}}-Q_{{\mathrm{DOX}}_{i}}}{Q_{{\mathrm{DOX}}_{0}}}\times 100\%,$$

where $Q_{{\mathrm{DOX}}_{0}}$ is the initial amount of DOX added to MBs/MCs and $Q_{{\mathrm{DOX}}_{i}}$ is the amount of DOX in the supernatant after incubation.

### 2.7. Analysis of the Kinetics of Doxorubicin Release

The release of DOX from MBs/MBs(+8L)/MCs(8L) was analyzed in a 0.05 M phosphate buffer solution (pH 6.0/7.4) at a temperature of 37 °C. Approximately 6 × 10^6^ MBs or MCs containing DOX and the same amount of placebo MBs/MBs(+8L)/MCs(8L) not containing DOX were placed into Eppendorf test tubes and suspended in 2.0 mL of the release medium. The samples were incubated under the conditions specified above, under constant stirring in a shaker (500 rpm). After fixed intervals of time (45 min, 1.5 h, 3 h, 6 h, 12 h, 24 h, and 48 h), the test tubes were centrifuged at 1900× *g* for 10 min at a temperature of 37 °C. The DOX content of the samples was estimated by means of spectrophotometry of the supernatant samples at the wavelength of the maximum DOX absorbance (485 nm). The proportion of DOX released from the microcarriers was calculated as:

(4) $$\mathrm{DOX}=\frac{A_{{\mathrm{DOX}}_{t}}}{A_{{\mathrm{DOX}}_{\mathrm{st}}}}\;\times \;100\%,$$

where $A_{{\mathrm{DOX}}_{t}}$ is the optical density of the release medium containing DOX at that moment, t, and $A_{{\mathrm{DOX}}_{\mathrm{st}}}$ is the optical density of the reference DOX solution in the release medium.

### 2.8. Statistical Analysis

The *Origin Pro*, version 8.5.0 SR1; Data Analysis and Graphing Software; OriginLab Corporation: Northampton, MA, USA, 2010 was used for statistical analysis. The data are presented as the mean values and standard deviations obtained from at least three experiments, if not indicated otherwise.

## 3. Results and Discussion

### 3.1. Obtaining Doxorubicin-Free and Doxorubicin-Containing Microbeads and Microcapsules with Different Structures

The synthesis of CaCO_3_ MBs using equimolar CaCl_2_ and Na_2_CO_3_ solutions in equivalent volumes yields the final product in the form of microparticles of the vaterite type (CaCO_3_ microparticles with a matrix structure) with a mean diameter from 3.8 to 6.5 μm [13]. For obtaining smaller microparticles with a narrow size distribution, thickening agents are added to the reaction mixture [12,16,37]. In the given case, the use of a water–glycerol mixture as a thickening agent ensures the stabilization of the vaterite polymorph formed and promotes a decrease in the CaCO_3_ MB size by facilitating the oversaturation of the reaction mixture and decreasing the rate of crystal formation [12,16,38].

**Figure 1:**
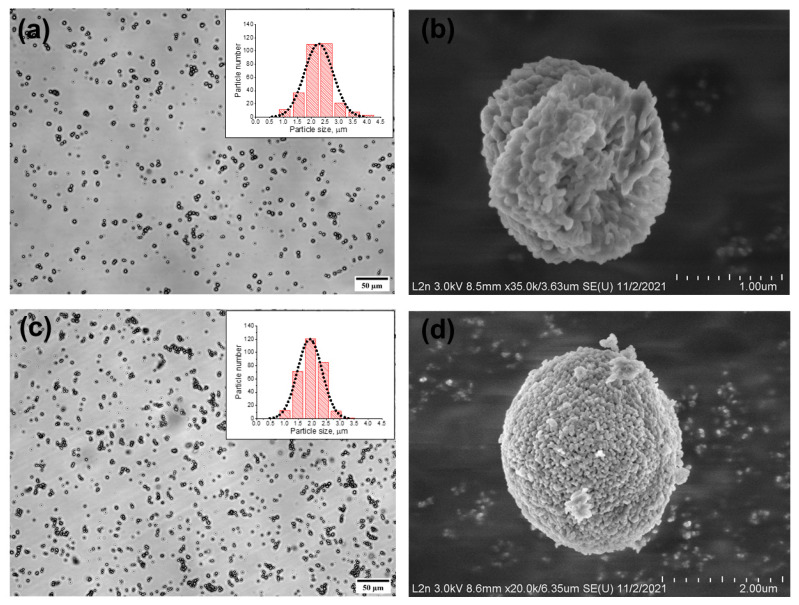
Microphotographs of typical doxorubicin-free and doxorubicin-containing calcium carbonate microbeads obtained by optical (**a**,**c**) and scanning electron (**b**,**d**) microscopies, respectively. The size distribution histograms of both particle types are presented in the insets (**a**,**c**).

The MBs obtained upon addition of the 44 wt % aqueous solution of glycerol to the reaction mixture at a 1:1 ratio between glycerol and the other components were characterized by a spherical shape and a narrow size distribution (Figure 1a). Table 1 shows the mean size of the obtained MBs. The prepared particles had a shape close to spherical and a porous structure, as seen in the SEM image (Figure 1b). Because vaterite is known to have a spherical shape and a porous inner matrix, with a particle diameter varying from 0.05 to 5 μm [16], we assume that the prepared particles represent precisely the vaterite crystalline form.

**Figure 2:**
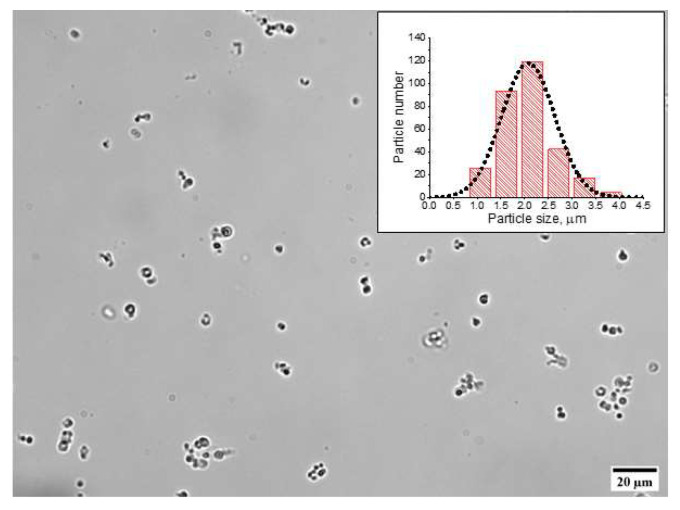
Microphotograph of typical polyelectrolyte microcapsules obtained by optical microscopy. The size distribution histogram of the prepared microcapsules is shown in the inset.

The polyelectrolyte shell was formed from PAH and PSS that had opposite charges, being a polycation and a polyanion, respectively (Table 2). The original MBs had a negative surface charge; therefore, the assembly of the polyelectrolyte shell began with adsorption of a polycation (PAH) to obtain PAH-coated MBs with a surface charge of 7.7 ± 2.2 mV. The subsequent application of a polyanion (PSS) layer yielded PAH/PSS-coated MBs with a negative surface charge of −18.6 ± 1.9 mV. Further successive adsorption of the polyelectrolytes was accompanied by a variation of the MB surface charge from +9.4 ± 0.5 to −31.6 ± 2.9 mV upon the adsorption of the polycation and polyanion, respectively, which confirmed the efficacy of the assembly of the MB polyelectrolyte shell. The formation of the (PAH/PSS)_4_ polyelectrolyte shell followed by dissolution of the core in the course of fabrication of the shell-type MCs(8L) did not cause significant changes in the mean size and the surface charge of the capsules (Table 1, Figure 2).

**Figure 3:**
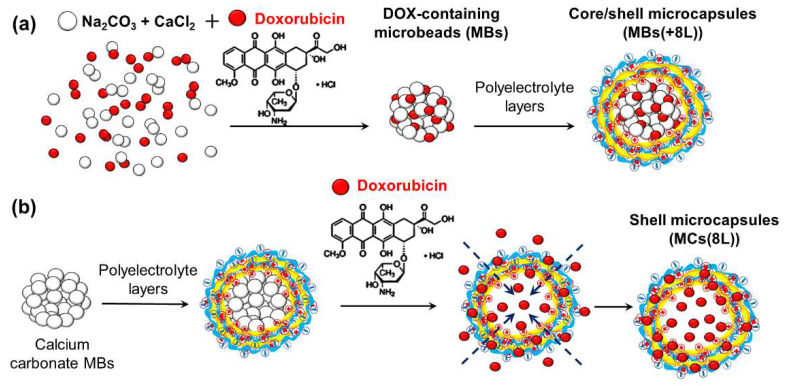
Schematic diagram of the preparation of doxorubicin-containing microcapsules. Two methods were used: (**a**) coprecipitation and (**b**) spontaneous loading. Abbreviations: MBs, microbeads; MCs, microcapsules; DOX, doxorubicin; 8L, polyelectrolyte layers (PAH/PSS)_4_ assembled at the microbead surface.

DOX can be loaded into the MCs at different stages of their preparation (Figure 3). Here, we determined the conditions of DOX incorporation into MBs in the course of their synthesis. One of the advantages of loading DOX at this stage is its incorporation directly into the vaterite matrix through its sorption during the formation of the MBs (Figure 3a). The synthesized DOX-containing CaCO_3_ MBs had the same spherical shape and porous structure as the DOX-free MBs and similar mean size (Figure 1c,d; Table 1).

The quantitative yield of the synthesis product was at least 3.5 × 10^9^ particles. The efficiency of drug encapsulation in the aqueous medium during synthesis was 73.1 ± 0.6%, which may be explained by the alkalization of the reaction mixture and the presence of the chloride (Cl^−^) counter-ions. Specifically, the pH value during the synthesis was about 9.8–9.9. All this suggests a decreased ionization of DOX hydrochloride salt, which may have caused a decrease in the DOX solubility and, hence, an increase in the rate of its incorporation into the MBs formed under the conditions of an oversaturated reaction mixture [35].

For obtaining core/shell MCs, we used the synthesized DOX-containing MBs, which were coated with the (PAH/PSS)_4_ shell. The resultant MBs(+8L) had a similar size (Table 1). In the course of the application of the polyelectrolyte shell, the surface charge of the particles varied from −32.6 ± 2.2 to +16.2 ± 1.3 mV, which also indicated effective assembly of the polyelectrolyte shell. The outermost polyelectrolyte layer consisted of PSS; therefore, the surface of the core/shell MCs was also negatively charged (Table 1).

Spontaneous loading of the drug into preliminarily prepared MCs under the conditions of changing permeability of the MC shell [15] is an alternative method for obtaining DOX-containing MCs(8L) with different structures. It can occur either via adsorption driven by electrostatic attraction between oppositely charged components of the system (in the given case, DOX with a positive charge of +21.3 ± 2.2 mV and PSS with a negative charge of −20.4 ± 2.2 mV) or via the diffusion of DOX into the polymer capsule shell upon changes in the pH and NaCl content of the MC microenvironment (Figure 3b). The resultant DOX-containing MCs(8L) had a weak negative charge compared to the original MCs because of the adsorption of DOX on their surface (Table 1). The mean size of the DOX-containing MCs(8L) did not differ significantly from that of DOX-free MCs. The efficiency of DOX encapsulation using this method was 65.6 ± 0.1%. Note that the efficiency of loading DOX into MCs with the same structure at room temperature was earlier reported to not exceed ~55% if the pH and ionic strength of the MC microenvironment remained unchanged [12]. The amount of DOX loaded by this method in our study may have been increased because, in the alkaline medium (pH 8.0) and in the presence of NaCl, the permeability of the polyelectrolyte shell was changed [39].

### 3.2. Functionalization of Microcapsules with Quantum Dots

**Figure 4:**
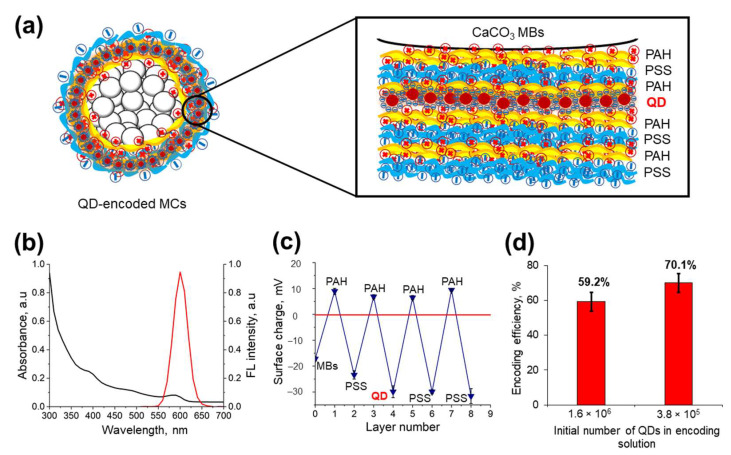
Obtaining microcapsules optically encoded with quantum dots (QDs). (**a**) A schematic diagram of the arrangement of the QD and PAH/PSS layers in the polyelectrolyte shell. (**b**) Optical characteristics of the CdSe/ZnS core/shell QDs solubilized with a PEG derivative (HS-(PEG)_12_-COOH). (**c**) Changes in the ζ-potential of the microbead/microcapsule surface upon application of the PAH/PSS polyelectrolytes and QD layers. (**d**) Estimated efficiency of the optical encoding of the microcapsules.

The polycation PAH and polyanion PSS used in this study contain, respectively, amine and sulfate groups that determine the electrostatic interaction between polymer layers, which results in the formation of interpolymer complexes and makes it possible to embed other electrically charged components [7,20,23]. The fluorescent MCs, optically encoded with water-soluble QDs, were fabricated by applying three layers of oppositely charged polyelectrolytes on the MB surface, following the order PAH/PSS/PAH, subsequently adsorbing the negatively charged QDs on the polyelectrolyte surface and coating the QD layer with PAH/PSS layers (Figure 4a).

The water-soluble QDs used for labeling the MCs were characterized by a wide light absorption spectrum and a narrow fluorescence spectrum with an emission peak at 590 nm (Figure 4b) and had a mean size (hydrodynamic diameter) of 32.3 ± 0.2 nm and a negative surface charge of −29.9 ± 2.3 mV. The PEG derivative (HS-(PEG)_12_-COOH) serving as a QD surface ligand determined the sufficient surface charge and colloidal stability of QDs in the aqueous phase, due to the presence of the carboxyl and thiol groups in the ligand structure. Molecules of the PEG derivative form a self-assembled monolayer at the nanocrystal surface. Thiol end groups of the PEG derivative have metal affinity and are oriented towards the QD shell consisting of zinc sulfide, while carboxylic groups are oriented away from the particle surface, thus enabling QD solubilization. The presence of the PEG chain prevents nanoparticle aggregation and enhances their colloidal stability [40].

This allowed the QDs to be efficiently adsorbed between the positively charged electrolyte layers in the course of encoding. Measurements of the MC surface charge after the application of the polyelectrolyte and QD layers showed that the changes in the ζ-potential were sufficient for the efficient adsorption of each successive layer (Figure 4c).

The efficiency of encoding was estimated by the amount of QDs adsorbed on the MC surface (Figure 4a). In our case, the optimal number of QDs in the solution for MC encoding was found to be 3.8 × 10^5^. A further increase in the number of QDs led to a decrease in the QD adsorption efficiency (Figure 4d). The photoluminescence properties of the water-soluble QDs embedded into the polyelectrolyte matrix may differ from that of the original QDs used in this study for microcapsule optical encoding. In particular, it was shown earlier that a slight red shift (up to 2 nm) of the fluorescence maximum occurs upon the immobilization of QDs of similar size and chemical composition between PAH layers [23,25]. However, the photoluminescence lifetime of the QDs encapsulated is characterized by a decay tendency resulting in average lifetime values of approximately ~8–9 ns, probably due to the charge transfer between the QDs and the polyelectrolyte microenvironment [13,23].

**Figure 5:**
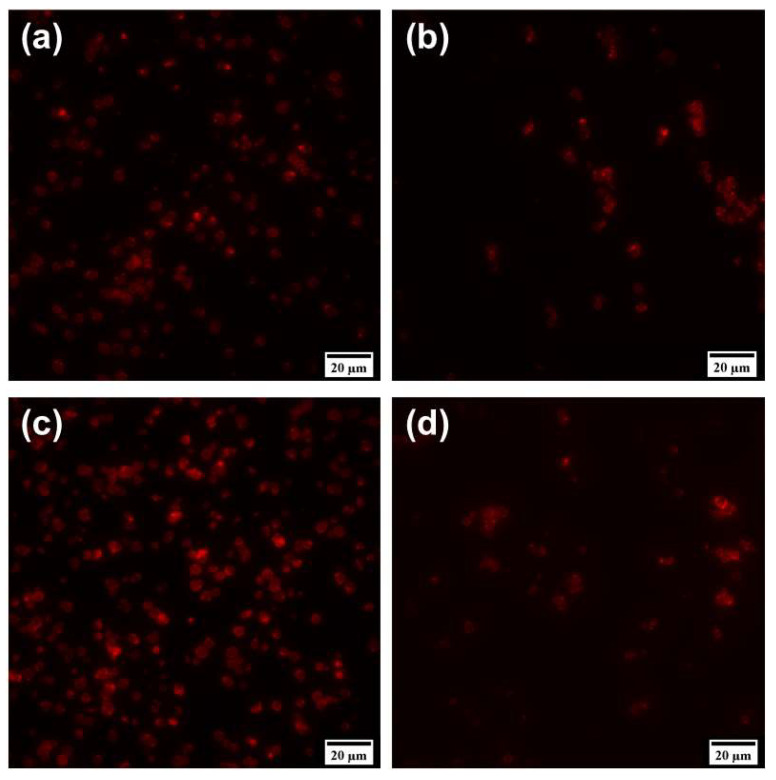
Fluorescence microscopy images of the polyelectrolyte microcapsules, optically encoded with quantum dots alone, (**a**) before, and (**b**) after dissolution of the calcium carbonate microbeads and dually doped both with quantum dots and doxorubicin, with (**c**) the drug encapsulated in the calcium carbonate microbeads and (**d**) the drug encapsulated into hollow microcapsules.

However, the resultant MCs, optically encoded with QDs, had a spherical or nearly spherical shape and were characterized by a bright fluorescence signal, as was demonstrated by means of fluorescence microscopy (Figure 5a,b). Analysis of the MC structure in the fluorescence mode showed that a cavity was left in the MCs after the core was dissolved, as evidenced by the greater transparency of MCs(8L) compared to MBs(+8L) (Figure 5b). Thus, the use of QDs for encoding MCs can make them suitable for subsequent fluorescence imaging [13,25,36]. Dual doping of MCs with both QDs and DOX also resulted in the formation of fluorescent particles (Figure 5c,d). The presence of the drug was not observed to alter the fluorescence properties of the engineered microstructures compared to MCs containing QDs alone.

### 3.3. Release of Doxorubicin from Microbeads and Microcapsules with Different Structures

**Figure 6:**
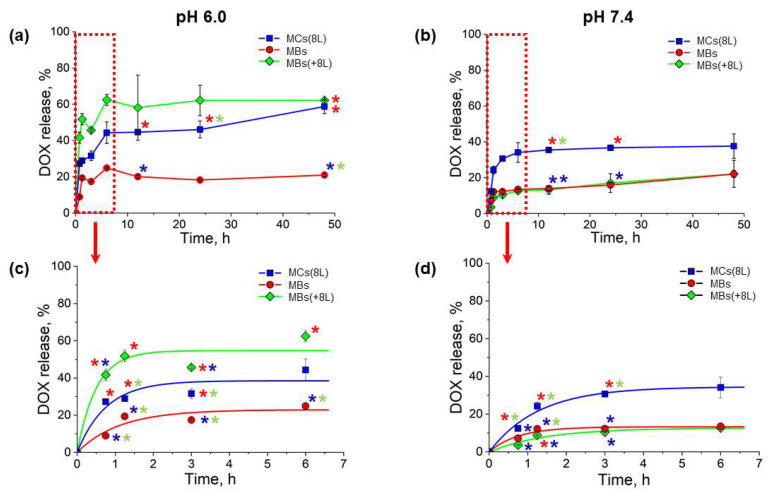
Profiles of doxorubicin release from microcapsules at (**a**) pH 6.0 and (**b**) pH 7.4 during 48 h and profiles of the initial drug release during the first 6 h of incubation at (**c**) pH 6.0 and (**d**) pH 7.4. Asterisks mark the data that significantly differ from one another (*p* < 0.05, Student’s *t*-test).

The release of DOX from the obtained MBs and MCs with different structures was analyzed under conditions mimicking those of the living body: a temperature of 37 °C and a pH value the same as that of normal tissues (7.4) or the local tumor environment (6.0) [17,18]. The results shown in Figure 6 demonstrate that the release of DOX from the MCs was prolonged at both pH values, 6.0 and 7.4, the cumulative DOX release within 48 h not exceeding 70%.

In a weakly acidic medium (pH 6.0), DOX was released at different rates from MBs, MBs(+8L), and MCs(8L). However, a burst release of DOX was observed during the first 2 h of incubation in all three cases (Figure 6a,c). The initial release pattern indicated that DOX was primarily released from the surface of MBs or MCs. Despite the presence of the (PAH/PSS)_4_ polyelectrolyte shell, the initial-burst DOX release was more intense in the case of MBs(+8L) compared to MBs. This may have been because the polyelectrolyte shell, including its outer layers, contained DOX as a result of its diffusion from the MB core into the interlayer space during the shell assembly. MCs(8L) exhibited a similar DOX release pattern. The lowest release rate was characteristic of DOX-containing MBs, apparently, because of the dissolution of DOX crystals on the MB surface, whereas MBs(+8L) and MCs(8L) contained DOX in the ionized form. In all cases, the release rate dropped after 3 h of incubation, which suggested that, now, DOX was released from within the microcarriers. After that, the release was further gradually decelerated.

At a pH of 7.4, DOX was released more slowly and evenly than at pH 6.0 (Figure 6b,d). The slower release may have resulted from a weaker ionization of DOX in the more alkaline medium. The initial release was less abrupt, especially in the cases of MBs(+8L) and MCs(8L). The intense-burst initial release of the drug from MBs and MBs(+8L) at pH 6.0 was also probably related to the surface erosion of the carrier matrix in weakly acidic media, as reported earlier [14,20]. It is noteworthy that the release of DOX from MCs(8L) was more intense compared to DOX-containing MBs and MBs(+8L). This could be accounted for by a higher permeability of the polyelectrolyte shell of the shell-type MCs because of a decreased charge density of PAH as a weak polyelectrolyte (with a pKa of about 8.5) [39]. Usually, when the core has been dissolved, the layers of the polyelectrolyte shell may be rearranged, which also alters the permeability of the polyelectrolyte structure, in contrast to core/shell MCs, where the shell, which is located on the core surface, is more stable [41]. However, the obtained data demonstrate that the small-sized MCs provide more extended DOX release compared to previously developed 5-μm alginate/chitosan MCs, which consist of 5 or 8 polyelectrolyte layers and are placed in an isotonic microenvironment or phosphate buffer saline (pH 7.4) [42,43]. The DOX release kinetics from calcium carbonate MBs was earlier shown to be pH-dependent and to be stimulated by MB erosion in a slightly acidic medium. The presence of additional polymers or proteins inside the MB structure (e.g., poly(vinyl sulfonate), dextran sulfate, and mucine) or at their surface also led to DOX release rate deceleration, preventing the possibility of an burst release of the drug [44,45]. 

## 4. Conclusions

In this study, we have developed size-homogenous MBs and MCs with different structures (core/shell and shell types), as well as various approaches to their functionalization with an antitumor drug (DOX) and their optical encoding with fluorescent nanolabels (water-soluble QDs). The encapsulation of DOX via coprecipitation at the stage of MB synthesis or spontaneous loading into prepared MCs(8L), in an alkaline medium in the presence of a counterion (Cl^−^), yields a sufficient degree of inclusion of the drug into the carriers. DOX release from MCs with different structures is prolonged under conditions mimicking those of the living body. The optical encoding of the MCs with QDs makes it possible to obtain brightly fluorescent MCs, which demonstrates the possibility of their use in fluorescence imaging. The results of the study have shown that the MCs developed can be used as efficient systems of delivery for low-molecular-weight antitumor agents. The obtained data pave the way to the further use of the designed MCs for in vitro tracking of the interaction between the particles and cancer cells by employing the fluorescence of the drug and QDs, as well as fluorescent monitoring of the drug release and drug accumulation within cell compartments upon their cellular uptake. The MCs engineered and presented herein have the potential to be used *in vivo* as a theranostic platform, providing simultaneous anti-cancer and fluorescent trafficking capacities.

## Figures and Tables

**Table 1:** Disperse characteristics of doxorubicin-free and doxorubicin-containing microbeads and microcapsules with different structures.

Sample *	Size, µm **	ζ-Potential, mV
DOX-free MBs	2.5 ± 07	−17.2 ± 0.8
DOX-free MCs	2.0 ± 0.4	−20.4 ± 2.2
DOX-containing MBs	2.0 ± 0.4	−12.5 ± 1.7
DOX-containing MCs(8L)	2.4 ± 0.6	−7.5 ± 2.2
DOX-containing MBs(+8L)	2.0 ± 0.8	−23.5 ± 2.4

* MBs, calcium carbonate microbeads; MCs, microcapsules; 8L, the number of assembled polyelectrolyte layers (PAH/PSS)_4_; DOX, doxorubicin; ** the size was estimated using optical microscopy. The microbeads and microcapsules with different structures did not differ significantly from one another in size (*p* > 0.05, Student’s *t*-test).

**Table 2:** Characteristics of the building blocks for the preparation of microcapsules.

Component *	Size, nm **	ζ-Potential, mV
PAH	13.9 ± 0.2	+15.9 ± 2.4
PSS	32.5 ± 2.8	−18.6 ± 1.9

* PAH, poly(allylamine hydrochloride); PSS, poly(sodium 4-styrene sulfonate); ** the size was determined as the hydrodynamic diameter by the dynamic light scattering method.

## Data Availability

Not applicable.

## References

[1] Chang D., Ma Y., Xu X., Xie J., Ju S. (2021). Stimuli-Responsive Polymeric Nanoplatforms for Cancer Therapy. Front. Bioeng. Biotechnol..

[2] Liu S., Lin T.-P., Li D., Leamer L., Shan H., Li Z., Gabbaï F.P., Conti P.S. (2013). Lewis Acid-Assisted Isotopic 18F-19F Exchange in BODIPY Dyes: Facile Generation of Positron Emission Tomography/Fluorescence Dual Modality Agents for Tumor Imaging. Theranostics.

[3] Kaufman G., Boltyanskiy R., Nejati S., Thiam A.R., Loewenberg M., Dufresne E.R., Osuji C.O. (2014). Single-step microfluidic fabrication of soft monodisperse polyelectrolyte microcapsules by interfacial complexation. Lab Chip.

[4] Nifontova G., Ramos-Gomes F., Baryshnikova M., Alves F., Nabiev I., Sukhanova A. (2019). Cancer Cell Targeting With Functionalized Quantum Dot-Encoded Polyelectrolyte Microcapsules. Front. Chem..

[5] Kolesnikova T.A., Kiragosyan G., Le T.H.N., Springer S., Winterhalter M. (2017). Protein A Functionalized Polyelectrolyte Microcapsules as a Universal Platform for Enhanced Targeting of Cell Surface Receptors. ACS Appl. Mater. Interfaces.

[6] Simioni A.R., De Jesus P.C.C., Tedesco A.C. (2018). Layer-by-layer hollow photosensitizer microcapsule design via a manganese carbonate hard template for photodynamic therapy in cells. Photodiagn. Photodyn. Ther..

[7] Novoselova M.V., Voronin D.V., Abakumova T., Demina P., Petrov A.V., Petrov V.V., Zatsepin T., Sukhorukov G.B., Gorin D.A. (2019). Focused ultrasound-mediated fluorescence of composite microcapsules loaded with magnetite nanoparticles: In vitro and in vivo study. Colloids Surf. B Biointerfaces.

[8] Pechenkin M.A., Möhwald H., Volodkin D.V. (2012). pH- and salt-mediated response of layer-by-layer assembled PSS/PAH microcapsules: Fusion and polymer exchange. Soft Matter.

[9] Trushina D.B., Bukreeva T.V., Borodina T.N., Belova D.D., Belyakov S., Antipina M.N. (2018). Heat-driven size reduction of biodegradable polyelectrolyte multilayer hollow capsules assembled on CaCO_3_ template. Colloids Surf. B Biointerfaces.

[10] Nifontova G., Kalenichenko D., Baryshnikova M., Gomes F.R., Alves F., Karaulov A., Nabiev I., Sukhanova A. (2019). Biofunctionalized Polyelectrolyte Microcapsules Encoded with Fluorescent Semiconductor Nanocrystals for Highly Specific Targeting and Imaging of Cancer Cells. Photonics.

[11] Zan X., Garapaty A., Champion J.A. (2015). Engineering Polyelectrolyte Capsules with Independently Controlled Size and Shape. Langmuir.

[12] Trushina D.B., Akasov R., Khovankina A., Borodina T., Bukreeva T.V., Markvicheva E.A. (2019). Doxorubicin-loaded biodegradable capsules: Temperature induced shrinking and study of cytotoxicity in vitro. J. Mol. Liq..

[13] Nifontova G., Zvaigzne M., Baryshnikova M., Korostylev E., Ramos-Gomes F., Alves F., Nabiev I., Sukhanova A. (2018). Next-Generation Theranostic Agents Based on Polyelectrolyte Microcapsules Encoded with Semiconductor Nanocrystals: Development and Functional Characterization. Nanoscale Res. Lett..

[14] Campbell J., Abnett J., Kastania G., Volodkin D., Vikulina A.S. (2021). Which Biopolymers Are Better for the Fabrication of Multilayer Capsules? A Comparative Study Using Vaterite CaCO3 as Templates. ACS Appl. Mater. Interfaces.

[15] Novoselova M.V., Loh H.M., Trushina D.B., Ketkar A., Abakumova T.O., Zatsepin T.S., Kakran M., Brzozowska A.M., Lau H.H., Gorin D.A. (2020). Biodegradable Polymeric Multilayer Capsules for Therapy of Lung Cancer. ACS Appl. Mater. Interfaces.

[16] Trushina D., Bukreeva T.V., Kovalchuk M.V., Antipina M.N. (2014). CaCO_3_ vaterite microparticles for biomedical and personal care applications. Mater. Sci. Eng. C.

[17] Bosio V.E., Cacicedo M.L., Calvignac B., León I., Beuvier T., Boury F., Castro G.R. (2014). Synthesis and characterization of CaCO _3_ –biopolymer hybrid nanoporous microparticles for controlled release of doxorubicin. Colloids Surf. B Biointerfaces.

[18] Boi S., Rouatbi N., Dellacasa E., Di Lisa D., Bianchini P., Monticelli O., Pastorino L. (2020). Alginate microbeads with internal microvoids for the sustained release of drugs. Int. J. Biol. Macromol..

[19] Souza E.F., Ambrósio J.A., Pinto B.C., Beltrame M., Sakane K.K., Pinto J.G., Ferreira-Strixino J., Gonçalves E.P., Simioni A.R. (2020). Vaterite submicron particles designed for photodynamic therapy in cells. Photodiagn. Photodyn. Ther..

[20] Volodkin D. (2014). CaCO_3_ templated micro-beads and -capsules for bioapplications. Adv. Colloid Interface Sci..

[21] Svenskaya Y., Garello F., Lengert E., Kozlova A., Verkhovskii R., Bitonto V., Ruggiero M.R., German S., Gorin D., Terreno E. (2021). Biodegradable polyelectrolyte/magnetite capsules for MR imaging and magnetic targeting of tumors. Nanotheranostics.

[22] Zharkov M.N., Brodovskaya E.P., Kulikov O.A., Gromova E.V., Ageev V.P., Atanova A.V., Kozyreva Z.V., Tishin A.M., Pyatakov A.P., Pyataev N.A. (2020). Enhanced cytotoxicity caused by AC magnetic field for polymer microcapsules containing packed magnetic nanoparticles. Colloids Surf. B Biointerfaces.

[23] Nifontova G., Krivenkov V., Zvaigzne M., Samokhvalov P.S., Efimov A.E., Agapova O.I., Agapov I.I., Korostylev E., Zarubin S., Karaulov A. (2020). Controlling Charge Transfer from Quantum Dots to Polyelectrolyte Layers Extends Prospective Applications of Magneto-Optical Microcapsules. ACS Appl. Mater. Interfaces.

[24] Demina P.A., Sindeeva O.A., Abramova A.M., Prikhozhdenko E.S., Verkhovskii R.A., Lengert E.V., Sapelkin A.V., Goryacheva I.Y., Sukhorukov G.B. (2021). Fluorescent Convertible Capsule Coding Systems for Individual Cell Labeling and Tracking. ACS Appl. Mater. Interfaces.

[25] Bilan R.S., Krivenkov V.A., Berestovoy M.A., Efimov A.E., Agapov I.I., Samokhvalov P.S., Nabiev I., Sukhanova A. (2017). Engineering of Optically Encoded Microbeads with FRET-Free Spatially Separated Quantum-Dot Layers for Multiplexed Assays. ChemPhysChem.

[26] Ramos-Gomes F., Bode J., Sukhanova A., Bozrova S.V., Saccomano M., Mitkovski M., Krueger J.E., Wege A.K., Stuehmer W., Samokhvalov P.S. (2018). Single- and two-photon imaging of human micrometastases and disseminated tumour cells with conjugates of nanobodies and quantum dots. Sci. Rep..

[27] Kage D., Hoffmann K., Nifontova G., Krivenkov V., Sukhanova A., Nabiev I., Resch-Genger U. (2020). Tempo-spectral multiplexing in flow cytometry with lifetime detection using QD-encoded polymer beads. Sci. Rep..

[28] Romoser A., Ritter D., Majitha R., Meissner K.E., McShane M., Sayes C.M. (2011). Mitigation of Quantum Dot Cytotoxicity by Microencapsulation. PLoS ONE.

[29] Song D., Cui J., Ju Y., Faria M., Sun H., Howard C.B., Thurecht K.J., Caruso F. (2019). Cellular Targeting of Bispecific Antibody-Functionalized Poly(ethylene glycol) Capsules: Do Shape and Size Matter?. ACS Appl. Mater. Interfaces.

[30] Xiong R., Hua D., Van Hoeck J., Berdecka D., Léger L., De Munter S., Fraire J.C., Raes L., Harizaj A., Sauvage F. (2021). Photothermal nanofibres enable safe engineering of therapeutic cells. Nat. Nanotechnol..

[31] Timin A.S., Lepik K.V., Muslimov A.R., Gorin D.A., Afanasyev B.V., Sukhorukov G.B. (2016). Intracellular redox induced drug release in cancerous and mesenchymal stem cells. Colloids Surf. B Biointerfaces.

[32] Ermakov A.V., Verkhovskii R.A., Babushkina I.V., Trushina D.B., Inozemtseva O.A., Lukyanets E.A., Ulyanov V.J., Gorin D.A., Belyakov S., Antipina M.N. (2020). In Vitro Bioeffects of Polyelectrolyte Multilayer Microcapsules Post-Loaded with Water-Soluble Cationic Photosensitizer. Pharmaceutics.

[33] Carvalho C., Santos R.X., Cardoso S., Correia S., Oliveira P.J., Santos M.S., Moreira P.I. (2009). Doxorubicin: The Good, the Bad and the Ugly Effect. Curr. Med. Chem..

[34] Mohan P., Rapoport N. (2010). Doxorubicin as a Molecular Nanotheranostic Agent: Effect of Doxorubicin Encapsulation in Micelles or Nanoemulsions on the Ultrasound-Mediated Intracellular Delivery and Nuclear Trafficking. Mol. Pharm..

[35] Tewes F., Munnier E., Antoon B., Okassa L.N., Cohen-Jonathan S., Marchais H., Douziech-Eyrolles L., Soucé M., Dubois P., Chourpa I. (2007). Comparative study of doxorubicin-loaded poly(lactide-co-glycolide) nanoparticles prepared by single and double emulsion methods. Eur. J. Pharm. Biopharm..

[36] Nifontova G., Efimov A., Agapova O., Agapov I., Nabiev I., Sukhanova A. (2019). Bioimaging Tools Based on Polyelectrolyte Microcapsules Encoded with Fluorescent Semiconductor Nanoparticles: Design and Characterization of the Fluorescent Properties. Nanoscale Res. Lett..

[37] Lu Z., Zhang J., Ma Y., Song S., Gu W. (2012). Biomimetic mineralization of calcium carbonate/carboxymethylcellulose microspheres for lysozyme immobilization. Mater. Sci. Eng. C.

[38] Vikulina A., Webster J., Voronin D., Ivanov E., Fakhrullin R., Vinokurov V., Volodkin D. (2020). Mesoporous additive-free vaterite CaCO3 crystals of untypical sizes: From submicron to Giant. Mater. Des..

[39] Musin E.V., Kim A.L., Tikhonenko S.A. (2020). Destruction of Polyelectrolyte Microcapsules Formed on CaCO_3_ Microparticles and the Release of a Protein Included by the Adsorption Method. Polymers.

[40] Bilan R., Fleury F., Nabiev I., Sukhanova A. (2015). Quantum Dot Surface Chemistry and Functionalization for Cell Targeting and Imaging. Bioconjugate Chem..

[41] Musin E.V., Kim A.L., Dubrovskii A.V., Tikhonenko S.A. (2021). New sight at the organization of layers of multilayer polyelectrolyte microcapsules. Sci. Rep..

[42] Tao X., Chen H., Sun X.-J., Chen J.-F., Roa W.H. (2007). Formulation and cytotoxicity of doxorubicin loaded in self-assembled bio-polyelectrolyte microshells. Int. J. Pharm..

[43] Zhao Q., Han B., Wang Z., Gao C., Peng C., Shen J. (2007). Hollow chitosan-alginate multilayer microcapsules as drug delivery vehicle: Doxorubicin loading and in vitro and in vivo studies. Nanomed. Nanotechnol. Biol. Med..

[44] Sudareva N., Suvorova O., Saprykina N., Vlasova H., Vilesov A. (2021). Doxorubicin delivery systems based on doped CaCO3 cores and polyanion drug conjugates. J. Microencapsul..

[45] Balabushevich N.G., Kovalenko E.A., Le-Deygen I.M., Filatova L.Y., Volodkin D., Vikulina A.S. (2019). Hybrid CaCO3-mucin crystals: Effective approach for loading and controlled release of cationic drugs. Mater. Des..

